# Enrichment of Circulating Tumor Cells from Whole Blood Using a Microfluidic Device for Sequential Physical and Magnetophoretic Separations

**DOI:** 10.3390/mi11050481

**Published:** 2020-05-06

**Authors:** Jusin Lee, Onejae Sul, Seung-Beck Lee

**Affiliations:** 1 Department of Electronic Engineering, Hanyang University, 222 Wangsimni-ro, Seongdong-gu, Seoul 04763, Korea; jusin19@hanyang.ac.kr; 2 Institute of Nano Science and Technology, Hanyang University, 222 Wangsimni-ro, Seongdong-gu, Seoul 04763, Korea; ojsul@hanyang.ac.kr

**Keywords:** microfluidics, cell separation, magnetophoresis, circulating tumor cell

## Abstract

Based on their high clinical potential, the isolation and enrichment of rare circulating tumor cells (CTCs) from peripheral blood cells has been widely investigated. There have been technical challenges with CTC separation methods using solely cancer-specific surface molecules or just using physical properties of CTCs, as they may suffer from heterogeneity or lack of specificity from overlapping physical characteristics with leukocytes. Here, we integrated an immunomagnetic-based negative enrichment method that utilizes magnetic beads attached to leukocyte-specific surface antigens, with a physical separation method that utilizes the distinct size and deformability of CTCs. By manipulating the pressure distribution throughout the device and balancing the drag and magnetic forces acting on the magnetically labeled white blood cells (WBCs), the sequential physical and magnetophoretic separations were optimized to isolate intact cancer cells, regardless of heterogeneity from whole blood. Using a breast cancer cell line in whole blood, we achieved 100% separation efficiency for cancer cells and an average of 97.2% for WBCs, which resulted in a 93.3% average separation purity. The experimental results demonstrated that our microfluidic device can be a promising candidate for liquid biopsy and can be a vital tool for aiding future cancer research.

## 1. Introduction

Since it has been reported that circulating tumor cells (CTCs) have crucial information about cancer and its metastasis, detecting and isolating CTCs means they can be utilized as diagnostic and prognostic biomarkers for cancer, and can assist in the research and treatments that consider the molecular characteristics of CTCs [1,2,3,4,5]. However, due to their intrinsic scarcity (1–100 cells/mL), diverse methods have been proposed to detect and isolate rare CTCs from a vast number of hematologic cells [6,7,8,9,10]. One of the most common approaches for CTC detection and isolation is positive enrichment, which directly uses an antigen-antibody relation with target specific proteins on CTCs, such as the epithelial cell adhesion molecule (EpCAM) [11,12,13,14,15]. However, this method suffers from heterogeneity of CTCs, and may lose its subpopulation when undergoing the epithelial-to-mesenchymal transition [16,17,18]. To overcome these issues, the use of other antibodies specific to certain cancer types or a mixture of antibodies has been proposed [19,20,21,22], but these methods need information about the specific cancer types or mutation in advance, and often require a high cost for visualizing the mixtures of antibodies. Furthermore, isolated CTCs can lose their characteristics after antibody binding, creating difficulties in further downstream analysis [23,24]. As an alternative separation method to using biomarkers, utilizing distinct physical characteristics of CTCs compared to normal blood cells, such as size, deformability, and higher stiffness, has been demonstrated [25,26,27,28]. However, the absence of selectivity still existed in relation to white blood cells (WBCs) with overlapping physical characteristics, to which the relatively low separation purity was attributed [25].

In this paper, we report on a microfluidic separation device that integrated an immunoaffinity-based negative enrichment method, which removed labeled WBCs with a physical separation method, and therefore isolated CTCs. By adding a method to remove WBCs with overlapping physical characteristics, it was possible to alleviate the required pressure difference levels for physical separation of CTCs, leading to higher levels of separation. We also analyzed the competition between the drag and magnetic forces acting on the magnetically labeled WBCs and were able to optimize the conditions to achieve their continuous removal.

## 2. Materials and Methods

### 2.1. Concept and Design

**Figure 1:**
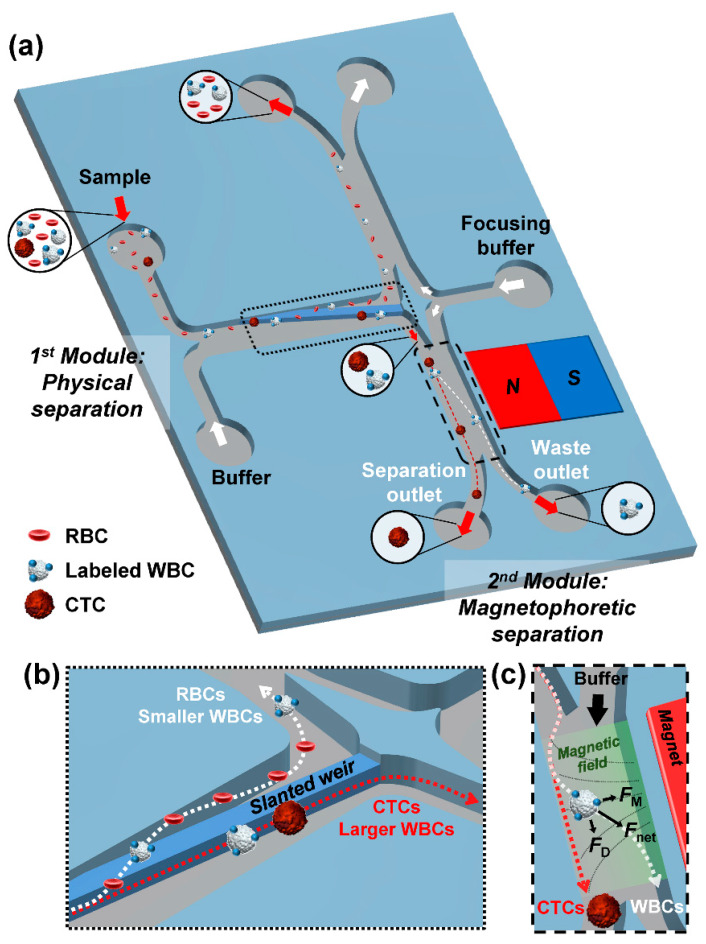
Schematic illustrations of the microfluidic separation device. (**a**) Overview of the enrichment sequence. Enlarged illustrations show (**b**) the physical separation in the first module, and (**c**) the magnetophoretic separation in the second module. Dotted arrows represent trajectories of cells.

The microfluidic device integrated modules for physical and magnetophoretic separations (Figure 1a). The first module had two inlets, for injection of the sample and the buffer solution, a slanted weir, and two outlets, which delivered separated cells to the second module and removed remaining cells to the waste outlet. The connected outlets of the first module had symmetric configurations, which were designed to maintain the pressure distribution along the first module. The second module had two inlets, one for injection of the separated cells from the first module and the other for the focusing buffer, a permanent magnet, and two outlets for waste and separated cell collection. The slanted weir traversed the length of the first module, from the upper wall of the main channel, to the middle of the fork that leads to the two outlets [25]. As shown in Figure 1b, most normal blood cells, which have smaller or comparable sizes to the weir gap (7 μm), would cross over the weir, while larger cells would be restricted and guided along to the second module. Since the weir-tracing larger cells experienced hydraulic pressure directed over the weir, it provided an additional separation criterion based on cell deformability, where highly deformable WBCs with sizes larger than the weir gap may have deformed and squeezed through. However, some smaller cancer cells may have also squeezed over the weir; therefore, maintaining the right pressure requirement became important. The slanted weir dimensions were optimized in our previous study (23 μm in height, 50 μm in width, and 0.8° angle) [25]. For continuous physical separation, the pressure drop along the weir (Δ*P*_x_) and across the weir (Δ*P*_y_) needed to have an appropriate ratio. We evaluated the device performance using COMSOL Multiphysics^®^ 5.4. (COMSOL Inc., Stockholm, Sweden). Here, we set the pressure drop ratio (Δ*P*_x_/Δ*P*_y_) to have a slightly larger value than one (Δ*P*_x_/Δ*P*_y_ = 1.1), based on previous experimental verifications. Furthermore, by decreasing the total flow rate of the first module, we alleviated Δ*P*_y_ to have 15 Pa and deformation-driven separation of cancer cells, which was the main cause of loss of cancer cells in the physical separation module. At the second module, a magnetic field was applied perpendicular to the fluid flow using a permanent magnet. As shown in Figure 1c, to prevent cancer cells from flowing to the waste outlet, all of the weir separated cells were focused and aligned to the far side of the channel, away from the magnet, by the focusing buffer. The magnetic field of the permanent magnet resulted in a magnetic force (*F*_M_), which depended on the number of magnetic beads attached to the WBCs. Meanwhile, since there was a drag force (*F*_D_) applied by the fluid flow, the focusing buffer flow rate needed to be optimized to have the net vector sum of the forces (*F*_net_) that directed the beaded WBCs towards the waste outlet, allowing the negative enrichment of CTCs.

### 2.2. Fabrication

The microfluidic device for sequential separations was fabricated on a 4.5 cm × 4.5 cm silicon chip (see Figure S1 for detail). To form a 7 μm weir gap for the physical separation module, double-layer photolithography was used with two different SU-8 negative photoresists (Microchem, Westborough, MA, USA). Before spin-coating, oxygen plasma (150 W, 100 mTorr, 60 s) was treated on the silicon chip to promote adhesion of the SU-8 photoresist. The first and second layers were spin-coated with SU-8 2050 and SU-8 2007 photoresists, with heights of 23 μm and 7 μm, respectively. The weir structure was patterned only on the first layer, and the other inlet, outlet, and main channel structures were patterned on both layers. The main channel dimensions of the first and second modules were both 500 μm in width, and 2 cm and 1 cm in length, respectively. For the encapsulation of the channel, a 5-mm-thick poly(dimethylsiloxane) (PDMS) plate was used to cover the patterned 4.5 cm × 4.5 cm chip with inlet and outlet holes and the void for the magnet defined. The patterned SU-8 chip was exposed to oxygen plasma (35 W, 100 mTorr, 30 s), and was then immersed into 5% (v/v) 3-aminopropyltriethoxysilane (APTES) in deionized water for 10 min at 80 °C. After rinsing with deionized water, it was bonded with the oxygen plasma (35 W, 100 mTorr, 30 s) treated PDMS plate on a 70 °C hot plate for 10 min under the applied pressure of ~5 N on the top.

### 2.3. Cell Preparation

To demonstrate the physical and magnetophoretic separation prior to the whole blood test, we prepared two different cell lines of HL-60 leukemia cells and MDA-MB-231 breast cancer cells, which express the CD45+ and CD45− phenotypes, respectively. HL-60 and MDA-MB-231 cells were purchased from the American Type Culture Collection (Manassas, VA, USA), and cultured in Dulbecco’s modified Eagle medium (DMEM) supplemented with 10% fetal bovine serum (FBS), penicillin (100 U/mL), and streptomycin (100 μg/mL) at 37 °C with 5% CO_2_. For the whole blood test, human peripheral blood was obtained from healthy donors at Hanyang University Seoul Hospital, with approval granted by the Hanyang University Institutional Review Board (HYI-17-057-5). All of the methods were carried out in accordance with the approved guidelines. HL-60 cells and WBCs were labeled with 4.5 μm Dynabeads CD45 (Invitrogen, Carlsbad, CA, USA), where the CD45 antigen is the leukocyte common antigen, which is expressed on almost all WBCs [29]. Taking into consideration that as much as 5 × 10^6^ WBCs exist in 1 mL of blood on average, 5 × 10^6^ HL-60 cells in 1 mL of 1× phosphate-buffered saline (PBS) solution were treated with 5 × 10^7^ beads in a tube for 30 min at 5 °C, with gentle tilting and rotation. To remove HL-60 cells without beads, we placed a magnet in the tube for 10 min and removed the supernatant. Then, the collected cells were resuspended in 1× PBS solution. Therefore, we assumed that the bead-less cells were removed and only bead-attached cells were considered. Furthermore, 1 mL of whole blood was treated with 5 × 10^7^ beads to magnetically label 5 × 10^6^ WBCs at the same condition as HL-60 cells.

### 2.4. Immunofluorescence

To assess the whole blood test, MDA-MB-231 cells were transduced with a green fluorescent protein (MSCV-IRES-eGFP) and spiked whole blood was used. For immunofluorescence microscopy after sequential separations, the collected cells from the separation outlet were centrifuged at 200 g for 5 min on a poly-l-lysine coated substrate. Immunofluorescent staining was performed using phycoerythrin (PE) conjugated anti-CD45 (Southern Biotech, Birmingham, AL, USA), and it was followed by counterstaining using 4′,6-diamidino-2-phenylindole (DAPI) (Vector Labs, Burlingame, CA, USA).

### 2.5. Magnetic Field Strength Measurement in the Fluidic Channel

For the magentophoretic separation module, a magnetic field was generated by a permanent neodymium magnet, and the magnetic field was applied perpendicular to the fluid flow. The magnetic field was measured by placing a 2 mm thick PDMS plate between the magnet and a teslameter, considering the device configuration and the experimental environment. The field strength was adjusted and measured by displacing the magnet away from the teslameter, as the magnetic field was proportional to the inverse square of the distance. Five average measured values of 3, 5.1, 8.1, 12.8, 20, and 25 mT from three measurements were used for the investigation of the effect of the magnetic force on cell separation. The difference in magnetic field strength perpendicular to fluid flow was ~0.4, 0.6, 0.9, 1.4, 2.2, and 2.8 mT between the fluidic wall positions for the magnetic field of 3, 5.1, 8.1, 12.8, 20, and 25 mT, respectively.

## 3. Results and Discussion

### 3.1. Demonstration of Sequential Separations Using Cell Lines

**Figure 2:**
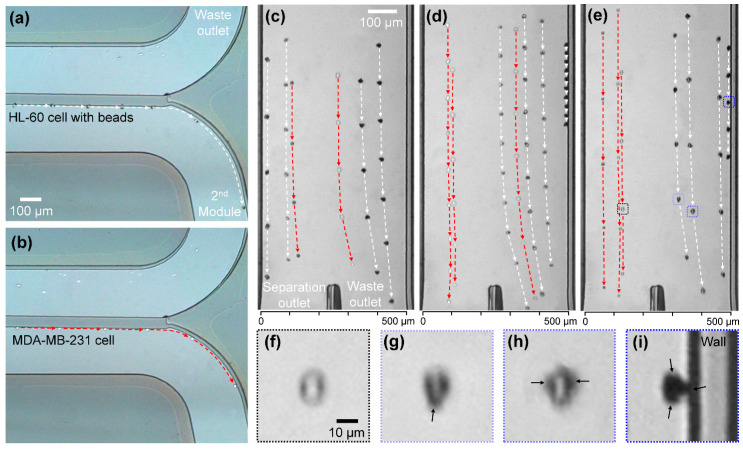
Demonstration of cell separation. (**a**) HL-60 cell and (**b**) MDA-MB-231 cell guided by the slanted weir in the physical separation module. HL-60 cells (white arrow) and MDA-MB-231 cells (red arrow) passing through the magnetophoretic separation module under various focusing buffer flow rates and magnetic field strength conditions of (**c**) 3 mL/h and 0 mT, (**d**) 3 mL/h and 20 mT, and (**e**) 5 mL/h and 20 mT. (**f**–**i**) Enlarged images of cells during separation from Figure 2e. (**f**) A separating MDA-MB-231 cell without a bead and separating HL-60 cells with (**g**) one bead (black arrow), (**h**) two beads, and (**i**) three beads.

In Figure 2, we present sequences of images that show the paths of the cells which were guided by the weir (Figure 2a–b) and separated by magnetophoresis (Figure 2c–e). One hundred MDA-MB-231 cells and 5 × 10^6^ HL-60 cells (magnetically labeled) in 1 mL of 1× PBS were introduced into the device, and the separated cells were counted at both outlets of the second module, to analyze their separation efficiencies. Due to the inherent varying degrees of the CD45 antigen expression levels and different cell sizes, the number of beads bound to HL-60 cells was distributed mostly from one to three, which existed in about 36%, 32%, and 32%, respectively, with few having more than four. Because the number of beads affects the separation efficiency, the flow rate and field strength should be optimized to enable all labeled cells to be continuously separated, regardless of the number of bound beads. For the first module, the total flow rate was set at 0.6 mL/h, and the sample-to-buffer flow ratio was fixed at 1:5, to assure high separation efficiency [25]. Most of the HL-60 cells flowed over the weir, which can be observed from the background images of Figure 2a–b, and these cells were swept away to the waste outlet. Meanwhile, the larger cells, which were mostly MDA-MB-231 cells and some larger HL-60 cells, followed along the slanted weir. The separation of an HL-60 cell with beads and an MDA-MB-231 cell are shown in Figure 2a–b, respectively, where sequential images taken at 0.25 s intervals were superimposed (see Movie S1).

The separated cells were delivered to the second module along the wall that extended from the weir, and the focusing buffer would flow parallel with them. In Figure 2c–e, the paths of the cells were tracked with time intervals of 3.3 ms, under different focusing buffer flow rates and magnetic field presence. For low flow rate (3 mL/h) without any magnetic field, the cells were commingled and went through both outlets without tendency (Figure 2c). When a magnetic field of 20 mT strength was applied, all labeled HL-60 cells were deflected and separated to the waste outlet, while MDA-MB-231 cells still passed randomly (Figure 2d). Then, when the focusing buffer flow rate increased to 5 mL/h, MDA-MB-231 cells were focused and led to the separation outlet with all labeled HL-60 cells deflected away (Figure 2e) (see Movie S2).

### 3.2. Effect of the Drag and Magnetic Forces on Cell Separation

**Figure 3:**
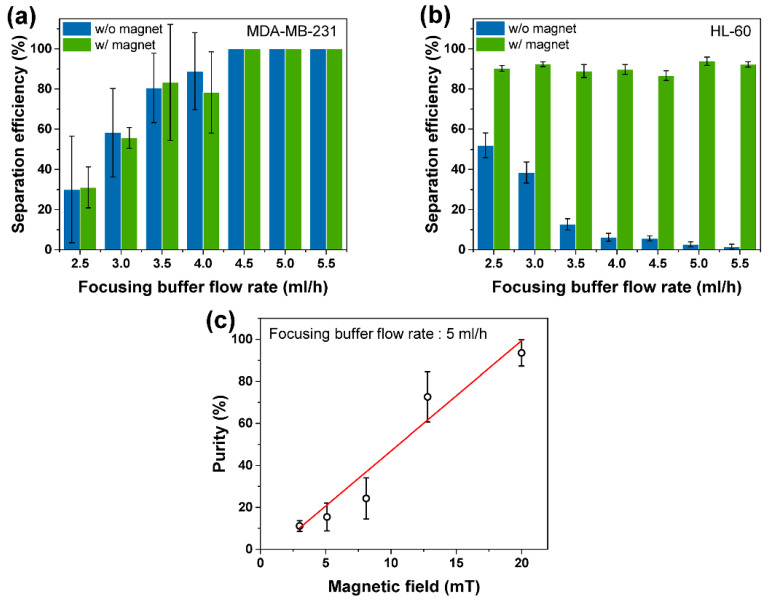
Separation efficiencies of (**a**) MDA-MB-231 cells and (**b**) HL-60 cells according to the focusing buffer flow rate. The applied magnetic field strength was 20 mT. (**c**) Purity at the separation outlet according to the magnetic field strength.

As mentioned, the deflection during magnetophoresis was influenced by the drag and magnetic forces experienced by the cells. The drag force was primarily governed by the size of the cells and the total flow rate [30]. The magnetic force was controlled by the magnetic field strength and depended on the number of beads bound to the cells [31]. Because the slanted weir separated and sorted cells by their size during the first stage, we assumed that the deflection angle would be attributed to the flow rate, the field strength, and the number of attached beads. To determine the appropriate flow rate, we performed quantitative analyses of the separation efficiencies of the cell types, according to the focusing buffer flow rate, which ranged from 2.5 to 5.5 mL/h, as shown in Figure 3a–b. Here, we obtained the separation efficiency by counting the separated cells visually using microscope images, which denoted the percentage of cells that were separated properly to the intended outlet. For MDA-MB-231 cells, regardless of the magnetic field, the separation efficiency increased with the flow rate and reached 100% for focusing buffer flow rates that were higher than 4.5 mL/h (Figure 3a). For HL-60 cells, with the applied magnetic field strength of 20 mT, most cells were separated to the waste outlet, showing high separation efficiency, with a maximum of 93.9% (Figure 3b). Without the magnetic field, the separation efficiencies decreased drastically in accordance with the increasing flow rates, indicating that most of the separated cells from the first module were focused to the separation outlet. We set the focusing buffer flow rate to 5.0 mL/h, which exhibited high efficiencies for both cell lines in the following experiments.

We further investigated the effect of the magnetic force on cell separation. The purity of the separated cancer cells according to the magnetic field strength was analyzed to determine the proper magnetic force (Figure 3c). We defined the purity as the ratio of cancer cells among the total separated cells collected from the separation outlet. The magnetic field strength of 20 mT resulted in the highest purity, with a maximum value of 93.5% on average. The cells bound by a single bead, to three or more beads showed different deflections, which made the cells have different lateral displacements at the second fork. The number of bound beads attached was counted visually using microscope images of the separated cells and lateral displacements were also measured. On average, the cancer cells (see example in Figure 2f) were aligned at a distance of 93 ± 29 μm away from the left wall, and away from the magnet. The cells bound with a single bead and two beads (see examples in Figure 2g–h, respectively) showed average lateral displacements of 335 ± 50 μm, and 415 ± 58 μm, respectively, from the left wall. On average, the applied magnetic field resulted in a minimum of ~123 μm perpendicular separation at the second module outlet between the cancer cells and the beaded WBCs, at the given flow rate and channel dimensions. Meanwhile, cells bound with three (see example in Figure 2i) or more beads showed higher deflection and encountered the wall with the magnet before reaching the fork, and were then seen to roll along the wall. When the magnetic field strength was gradually decreased to 3 mT by displacing the magnet away from the fluidic wall, the separation purity was reduced from 93.5% to 11.1%. This demonstrated that the drag force became more dominant as the magnetic force was reduced below 20 mT at the given flow rate. On the other hand, when the field strength was increased above 20 mT, some labeled cells became trapped, due to higher friction at the walls by the higher magnetic field, and agglomerated together, reducing waste cell collection and lowering separation efficiencies (see Movie S3).

### 3.3. Separation of Spiked Cancer Cells From Whole Blood

**Figure 4:**
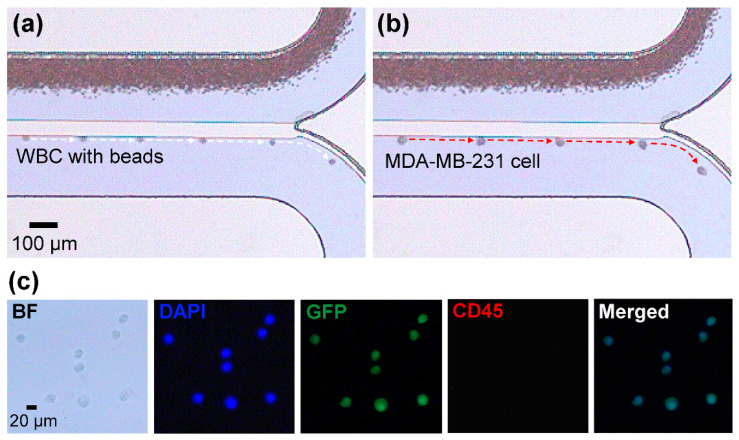
Physical separation of (**a**) a WBC and (**b**) an MDA-MB-231 cell from whole blood by the slanted weir. (**c**) Bright field and fluorescence images of MDA-MB-231 cells collected from the separation outlet. Cancer cells were distinguished from WBCs by immunofluorescent staining of DAPI for nucleus, GFP for cancer, and CD45 for WBC. The cells that express DAPI+/GFP+/CD45− phenotype were considered to be MDA-MB-231 cells.

Based on the demonstration of the basic operation of the device, the capability to process whole blood was tested using 1 mL of magnetically treated whole blood spiked with one hundred MDA-MB-231 breast cancer cells. To evaluate the performance, the flow rates for both modules and the magnetic field strength were fixed at the conditions showing the highest separation efficiencies and purity (the flow rates for the blood sample, buffer, and focusing buffer were 0.1, 0.5, and 5 mL/h, respectively, and the field strength was 20 mT). Figure 4a–b show the separated cells guided by the weir (see Movie S4). The background images of Figure 4a–b show that most red blood cells (RBCs) and WBCs flowed over the weir and were guided towards the waste outlet. The larger and less deformable cells, mostly expected to be MDA-MB-231 cells and some WBCs, rolled along the weir and were delivered to the following separation module. MDA-MB-231 cells were not present among the wasted cells from the waste outlet of the first module, when observed by immunofluorescence microscopy for GFP expression. Therefore, we could assume that all injected cancer cells were guided along the weir with a recovery ratio of 100% for the first physical separation module. When we observed all weir separated cells at the second module, the proportion of cancer cells was 6%, which was the purity of the physical separation module.

For the whole blood test, it was difficult to visually confirm whether magnetic beads adhered to WBCs during cell preparation due to the presence of red blood cells. Because of the difference with HL-60 cells, we chose immunofluorescence microscopy for evaluation of the whole blood test. Immunofluorescence microscopy, as well as image analysis, were performed on the cells collected from the separation outlet (Figure 4c), which showed that they only consisted of MDA-MD-231 cells. The separation efficiencies for the blood sample followed the results demonstrated earlier, showing 100% for MDA-MB-231 cells and an average of 97.2% for WBCs, with 93.3% purity. The separation efficiency of 100% for MDA-MB-231 cells denotes that all injected cancer cells through the inlet were properly separated and collected in the separation outlet. In total, of 5.6 mL of the solution containing 0.1 mL of the whole blood sample, a quarter (1.4 mL) came out from the separation outlet. From three whole blood tests, the number of collected cancer cells was 14 on average, so we can infer that the cancer cell concentration in the separation outlet was 10 cells/mL.

Using the separated cells from the separation outlet, we further evaluated the enrichment yield. Compared with the sole weir-based physical separation device, the integrated device displayed a higher cancer cell enrichment yield, defined as the ratio of cancer cells to WBCs after the separation, divided by the ratio before the separation. Although the weir-based physical separation enriched the cancer cells with the yield of 3.2 × 10^3^ on average, the integrated separation achieved yields of 7 × 10^5^ on average, which indicated that the integrated separations improved the enrichment by up to 219-fold.

## 4. Conclusions

In summary, integration of the physical separation method based on the size and deformability with immunomagnetic-based separation enabled effective isolation of intact cancer cells with high selectivity. By analyzing the relationship between drag and magnetic forces on magnetically labeled cells, our device isolated cancer cells from undiluted whole blood, achieving high separation efficiency, purity, and enrichment yield. From the results, we expect that this technique can be applied to liquid biopsy and to future cancer research based on separated CTCs.

## Supplementary Materials

The following are available online at https://www.mdpi.com/2072-666X/11/5/481/s1, Figure S1: CAD drawing of the sequential microfluidic separation device, Movie S1: Demonstration of physical separation using cell lines (HL-60 and MDA-MB-231), Movie S2: Demonstration of magnetophoretic separation using cell lines (HL-60 and MDA-MB-231), Movie S3: Agglomeration of magnetically labeled cells by magnetic field (HL-60), Movie S4: Demonstration of physical separation using whole blood (MDA-MB-231 spiked whole blood).
